# Antibacterial, antifungal and antioxidant activities of the ethanol extract of the stem bark of *Clausena heptaphylla*

**DOI:** 10.1186/1472-6882-12-232

**Published:** 2012-11-27

**Authors:** Md Fakruddin, Khanjada Shahnewaj Bin Mannan, Reaz Mohammad Mazumdar, Hafsa Afroz

**Affiliations:** 1 Industrial Microbiology Laboratory, Institute of Food Science and Technology (IFST). Bangladesh Council of Scientific and Industrial Research (BCSIR), Dhaka, Bangladesh; 2 Center For Food & Waterborne Diseases, icddr,b, Dhaka, Bangladesh; 3 BCSIR Laboratories- Chittagong, Chittagong, Bangladesh; 4 Department of Microbiology, Primeasia University, Dhaka, Bangladesh

**Keywords:** Antioxidant, Antibacterial, Antifungal, Cytotoxic, *Clausena heptaphylla*

## Abstract

**Background:**

There is wide spread interest in drugs derived from plants as green medicine is believed to be safe and dependable, compared with costly synthetic drugs that have adverse effects.

**Methods:**

We have attempted to evaluate the antioxidant, *In vitro* thrombolytic, antibacterial, antifungal and cytotoxic effects of *Clausena heptaphylla* (Rutaceae) stem bark extract ethanol extract.

**Results:**

Ethanolic stem bark extract of *Clausena heptaphylla* (CHET) contains flavonoids, alkaloids, saponins and steroids but it lacks tannins, anthraquinones and resins. Phenol content of the extract was 13.42 mg/g and flavonoid content was 68.9 mg/g. CHET exhibited significant DPPH free radical scavenging activity with IC_50_ value of 3.11 μg/ml. Reducing power of CHET was also moderately stronger. In the cytotoxicity assay, LC_50_ and Chi-square value of the ethanolic extract against brine shrimp nauplii were 144.1461 μg/ml and 0.8533 demonstrating potent cytotoxic effect of the extract. *In vitro* thrombolytic activity of CHET is significant with 45.38% clot lysis capability compared to that of Streptokinase (65.78%). In antibacterial screening, moderate zone of inhibition (6.5-9.0 mm in diameter) was observed against gram-positive *Bacillus subtilis* ATCC 11774*, Bacillus cereus* ATCC 10876*, Staphylococcus aureus* ATCC 25923*, Bacillus polymyxa* ATCC 842 *and Bacillus megaterium* ATCC 13578 and less promising zone of inhibition (3.0-4.5 mm in diameter) against gram-negative *Salmonella typhi* ATCC 65154*, Shigella flexneri* ATCC 12022*, Proteus vulgaris* ATCC 13315 *and Escherichia coli* ATCC 25922*. Shigella sonnei* ATCC 8992 did not show any sensitivity*.* The MIC values against these bacteria were ranged from 2,000 to 3,500 μg/ml. The extract showed significant zone of inhibition against *Rhizopus oryzae* DSM 2200*, Aspergillus niger* DSM 737 *and Aspergillus ochraceus* DSM 824 in antifungal assay*.*

**Conclusions:**

Further advanced research is necessary to isolate and characterize the chemical components responsible for the therapeutic properties of the plant.

## Background

Medicinal plants are an important therapeutic aid for various ailments [1]. Most of the people in rural and urban areas of the world depended on the medicinal plants for the treatment of infectious diseases [2].Today, there is wide spread interest in drugs derived from plants. Medicinal plant extracts offer considerable potential for the development of new agents effective against infections currently difficult to treat [3]. As natural products have been elaborated within living systems, they are often perceived as showing more ‘drug likeness’ and biological friendliness than totally synthetic molecules, making them good candidates for further drug development [4].

*Clausena heptaphylla* (Roxb.) Wight and Arn. (Bengali name: Panbilash, Karanphul, Pomkaphur) is a small bushy shrub that is distributed throughout Bangladesh, India and other parts of south East Asia [5]. *Clausena* species are known to be useful in paralysis, ulcerated nose, headache, muscular pain and malarial fever [6]. They are also reputed as diuretic, astringent, insecticide, tonic and vermifuge [7]. The leaves of the plants possess antimicrobial properties [8,9]. A new carbazole alkaloid, named clausenal, was isolated from the leaves of *C. heptaphylla* and the alkaloid was found to be active against both Gram-positive and Gram-negative bacteria and fungi [10].

We have attempted to evaluate the antioxidant, *In vitro* thrombolytic, antibacterial, antifungal and cytotoxic effects of *C. heptaphylla* stem bark ethanolic extract in this study.

## Methods

### Ethical statement

All animal experiments were carried out on shrimp nauplii, for which ethical approval is not required. All human blood samples were obtained from consenting, healthy individuals. There are no ethical review bodies in Bangladesh, so it was not possible for us to obtain approval for this procedure.

### Media and chemicals

DPPH (2,2-diphenyl-1-picrylhydrazyl), TCA (trichloroacetic acid) and ferric chloride were obtained from Sigma Chemical Co. USA; Ascorbic acid was from SD Fine Chem. Ltd. India, ammonium molybdate from Merck, Germany. Mueller-Hinton broth and agar media (Hi media, India), final PH 7.3±0.2 (at 25^º^C), was used for MIC determination and antibacterial. On the other hand, potato dextrose agar media (Hi media, India), final PH (at 25^º^C) 5.6±0.2 and artificial seawater (3.8% NaCl solution) were used for the determination of antifungal and cytotoxic effect [1].

### Collection of plant material

The stem bark of *C. heptaphylla* was collected from Jhalokathi, Bangladesh in june 2011 and was identified. The plant was identified by Sarder Nasir Uddin (Taxonomist, Bangladesh National Herbarium, Ministry of Environment and Forest, Dhaka, Bangladesh). A voucher specimen is preserved in Bangladesh National Herbarium with the accession No. DACD- 52876.

### Extraction of plant material

The fresh stem barks of *C. heptaphylla* were washed with water immediately after collection. Then chopped into small pieces, air dried at room temperature for about 10 days and pulverized into powder (1 kg) which was macerated in 6 L pure ethanol for 7 days at room temperature (23±5)^º^C. After 7 days, extract was filtered off through cotton plug and finally with a Whatman No. 1 filter paper. Then extract was concentrated under reduced pressure below 50^º^C through rotatory vacuum evaporator (RE200 Sterling, UK). The concentrated extract (45 gm blackish-green, 4.5% w/w yield) was stored at 4^º^C [11].

### Phytochemical screening

To identify the chemical constituents of plant extract standard procedures were followed. Freshly prepared crude extracts of *C. heptaphylla* were qualitatively tested for the presence of chemical constituents using the following reagents and chemicals, flavonoids with the use of Mg and HCl; tannins with ferric chloride and potassium dichromate solutions and saponins with ability to produce stable foam and steroids with Libermann Burchard reagent, reducing sugars with Benedict’s reagent and observed color change in respective [12].

### Determination of total phenolic content

Folin-Ciocalteu method was used to determine the total phenolic content; Folin-Ciocalteu oxidized the extract whereas sodium carbonate neutralized it. Blue color formed and the absorbance was measured at 760 nm after 60 min by using gallic acid (GA) as standard. Total Phenolic content was expressed as mg GA equivalent/gm of extract [13].

### Determination of total flavonoid content

Method described by Meda et al. [14] was followed to determine the flavonoid content where quercetin was used as standard. 1 mg of plant extract in methanol was mixed with 1 ml of aluminium trichloride in Ethanol (20 mg/ml) and a drop of acetic acid was added. Then diluted up to 25 ml with ethanol and measured the absorbance at 415 nm after 40 min. The absorption of blank samples and standard quercetin solution (0.5 mg/ml) in methanol was measured under the same conditions [14].

### DPPH radical scavenging activity

The antioxidant activity of *C. heptaphylla* ethanolic stem bark extract and the standard antioxidant ascorbic acid was assessed on the basis of the radical scavenging effect of the stable 2,2- diphenyl-1-picrylhydrazyl (DPPH) free radical activity according to the method described by Brand-Williams *et al.*[15]. *C. heptaphylla* ethanolic extract with different concentrations (10, 50, 100, 200, 400, 600, 800 and 1000 μg/ml) were prepared in ethanol. Ascorbic acid was used as standard in 1- 100 μg/ml solution. The scavenging activity against DPPH was calculated using the following equation: Scavenging activity (%) = [(A-B)/A] x100, Where A was the absorbance of control (DPPH solution without the sample), B was the absorbance of DPPH solution in the presence of the sample (extract/ascorbic acid). Then, % scavenging activity was plotted against log concentration and from the graph IC_50_ (Inhibition concentration 50) value was calculated by linear regression analysis.

### Reducing power

The reducing power of *C. heptaphylla* extract was determined according to the method of Oyaizu [16]. Different concentration of *C. heptaphylla* extract in 1ml of distilled water was mixed with phosphate buffer (2.5 ml, 0.2 M, pH 6.6) and potassium ferricyanide [K_3_Fe(CN)_6_ (2.5 ml, 1%) then the mixture was incubated at 50°C for 20 min. Trichloroacetic acid (10%) slightly added (2.5 ml)to the mixture and centrifuged at 3,000 rpm for 10min. The upper layer of the solution (2.5 ml) was mixed with distilled water (2.5 ml) and FeCl_3_ (0.5 ml, 0.1%) then taken the absorbance at 700 nm. The reference standard was Ascorbic acid and the Blank solution contained Phosphate buffer [16].

### Brine shrimp lethality bioassay

Cytotoxic activity of ethanolic stem bark extract was determined by Brine-Shrimp Lethality assay as described by Meyer *et al.*[17]. Simple zoological organism (*Artemia*) was used as a convenient monitor for the screening. The eggs of the brine shrimp were hatched in artificial seawater (3.8% NaCl solution) for 48 hours to mature shrimp called nauplii. Then 30 mg of *C. heptaphylla* stem bark extract were separately dissolved in 3 ml of DMSO, and from these 1000, 500, 250, 125 and 62.5 μg/ml were prepared by serial dilution. Each concentration was tested in triplicate, giving a total of 15 test tubes for each sample. A control containing 5 ml of DMSO solvent was used for each solvent. The final volume of the solution in each test-tube was made up to 5 ml with seawater immediately after adding shrimp larvae. The test tubes were maintained under illumination. After 24 hours have elapsed, Survivors were counted with the aid of a 3x magnifying glass. The LC_50_ values were calculated from Probit Chart using computer software “BioStat-2007”.

### *In vitro* thrombolytic activity

Individuals from whom the blood samples were collected were informed and upon their consent they were included in this study. Samples were taken from adult healthy individual. No children were included in this study. *In vitro* thrombolytic activity was performed according to Ratnasooriya *et al.*[18].The extract was suspended in 10 ml distilled water and shaken vigorously on a vortex mixer. Then the suspension was kept overnight and decanted to remove the soluble supernatant, which was filtered through a filter paper (Whatman No. 1). The solution was then ready for *in vitro* evaluation of clot lysis activity [19]. Commercially available lyophilized Streptokinase vial (Polamin Werk GmbH, Herdecke, Germany) of 15,00,000 I.U., 5 ml sterile distilled water was added and mixed properly. This suspension was used as a stock from which 100 μl (30,000 I.U) was prepared for *in vitro* thrombolysis. Experiments for clot lysis were carried as reported earlier (Prasad *et al.*, 2007). Venous blood drawn from healthy volunteers was transferred in different pre-weighed sterile eppendorf tube (500 μl/tube) and incubated at 37°C for 45 minutes. After clot formation, serum was completely removed (aspirated out without disturbing the clot formed). Each tube having clot was again weighed to determine the clot weight (Clot weight = weight of clot containing tube – weight of tube alone). Each eppendorf tube containing clot was properly labeled and 100 μl of plant extract was added to the tubes. All the tubes were then incubated at 37°C for 90 minutes and observed for clot lysis. After incubation, fluid obtained was removed and tubes were again weighed to observe the difference in weight after clot disruption. Difference obtained in weight taken before and after clot lysis was expressed as percentage of clot lysis. Streptokinase and water were used as a positive and negative (non-thrombolytic) control respectively. The experiment was repeated several times with the blood samples of different volunteers. Clot lysis was calculated by the following formula:

$$\begin{array}{l} \text{\%clotlysis}=\left(\text{Weightofthelysisclot}/\right)\\ \;\left(\text{Weightofclotbeforelysis}\right)\times 100 \end{array}$$

### Antibacterial assay

*In vitro* antibacterial screening was carried out by disc diffusion method [20] against 6 gram-positive (*Bacillus subtilis, Staphylococcus aureus, Bacillus cereus, Bacillus polymyxa, Bacillus megaterium, Enterococcus faecalis*) and 8 gram-negative bacteria (*Salmonella typhi, Klebsiella sp., Shigella flexneri, Shigella sonnei, Proteus sp., E. coli, Vibrio cholerae, Pseudomonas aeruginosa*). The bacterial suspension turbidity adjusted to McFarland standard number 0.5, in Mueller Hinton Broth (Himedia, India). With a sterile cotton swab bacterial culture was streaked on previously prepared Mueller Hinton agar plate (Himedia, India). Dried and sterilized paper discs were treated separately with desired concentration of previously prepared ethanolic solution of the root extract using a micropipette dried in air under aseptic condition and placed at equidistance in a circle on the seeded plate. The concentrations of root extract used were 2 mg/disc and 3 mg/disc. These plates were kept for 4-6 hours at low temperature and the test materials diffuse from disc to the surrounding medium by this time. The plates were then incubated at 37°C for 18 hours. The diameter of zone of inhibition produced by root extract was then compared with standard antibiotic Kanamycin (30 μg/disc). Each sample was used in triplicate for the determination of antibacterial activity. Blank disc impregnated with solvent ethanol followed by drying off was used as negative control.

### Determination of MIC

Minimum inhibitory concentrations (MIC) of crude extract of the *C. heptaphylla* were performed by macrodilution method [21]. The crude extract was dissolved in 30% dimethyl sulfoxide (DMSO) to obtain 10% (w/v) solution. For MIC test of the selected bacteria, the extract was first diluted in sterilized Mueller-Hinton broth to the highest concentration of 10,000 μg/ml and then dilution were performed at concentration of 5000 μg/ml, 4000 μg/ml, 3500 μg/ml, 3000 μ/ml, 2500 μ/ml, 2000 μg/ml, 1500 μg/ml, 1000 μg/ml, 750 μg/ml, 500 μg/ml and 250 μg/ml in screw caped tube containing broth medium. Bacterial suspensions of the test organism were prepared in sterilized Mueller-Hinton broth. Then 1 ml of the dilution was added to each sterilized screw capped tube containing 1 ml of compound suitably diluted in the sterilized broth medium to give final volume of 2 ml. Culture medium without samples and others without microorganisms were used in the tests as control. Tubes were incubated at 37^0^C for 20-24 hours and growth was indicated by turbidity.

### Antifungal assay

The poisoned food technique [22] was used to screen for antifungal activity. Potato dextrose agar (PDA) was used as a culture medium. From this required concentration of extract was taken by sterilized pipette in a sterilized petriplate and then 15 ml medium was poured into the petriplate and mixed well and allowed to solidify. Inoculation was done at the center of each plate with 5 mm mycelium block for each fungus. The mycelium block was prepared with the help of cork borer from the growing area of a 5 days old culture of the test fungi on PDA. The blocks were placed at the center of each petriplate in an inverted position to get greater contact of the mycelium with the culture medium. The inoculated plates were incubated at (25±2)^0^C. Proper control (PDA without extract) was also maintained. Diameter of fungal colonies was measured after 5 days of incubation. The average of triplicate of measurements was taken as colony diameter of the fungus in millimeters. The percentage inhibition of mycelial growth of the test fungus was calculated by the following formula:

$$\text{I=}\left(\text{C-T}\right)\text{/C×}100$$

where, I=Percentage of Inhibition, C=Diameter of the fungal colony in control, T=Diameter of the fungal colony in treatment [11].

### Statistical analysis

All the *in vitro* experimental results were given as mean± SEM of three parallel measurements and data were evaluated by using student’s t-test. P values <0.001 were regarded as significant. Results were processed by Excel (2007) and BioStat [23,24].

## Results

### Phytochemical screening

Qualitative Phytochemical tests of *Clausena heptaphylla* were performed for the ethanol extract of the bark of stem. The results of various chemical tests for the detection and identification of chemical constituents were summarized in Table 1. The table indicates the presence of some chemical constituents present in the plant parts.

**Table 1:** **Result of phytochemical screening of ethanol stem bark extract of *C. heptaphylla***

**Tests**	**CHET**
Flavonoid	+
Tannin	-
Glycoside	+
Alkaloid	+
Anthraquinone	-
Carbohydrate	+
Resin	-
Protein	-
Saponins	+
Steroids	+

(CHET denote for ethanolic extracts of *Clausena heptaphylla;* (+) = present; (-)= Absent).

### Total phenol and flavonoid content

The total phenol and total flavonoid contents of *Clausena heptaphylla* of ethanol extract were expressed in Gallic acid and Quercetin equivalents respectively. Total phenolic content was 13.42 mg/g Gallic acid equivalent and total flavonoid content was 68.9 mg/g Quercetin equivalent.

Different studies suggest that different types of polyphenolic compounds such as flavonoids, phenolic acids which are found in plants have multiple biological effects, including antioxidant activity [25].

### DPPH free radical scavenging activity

**Figure 1:**
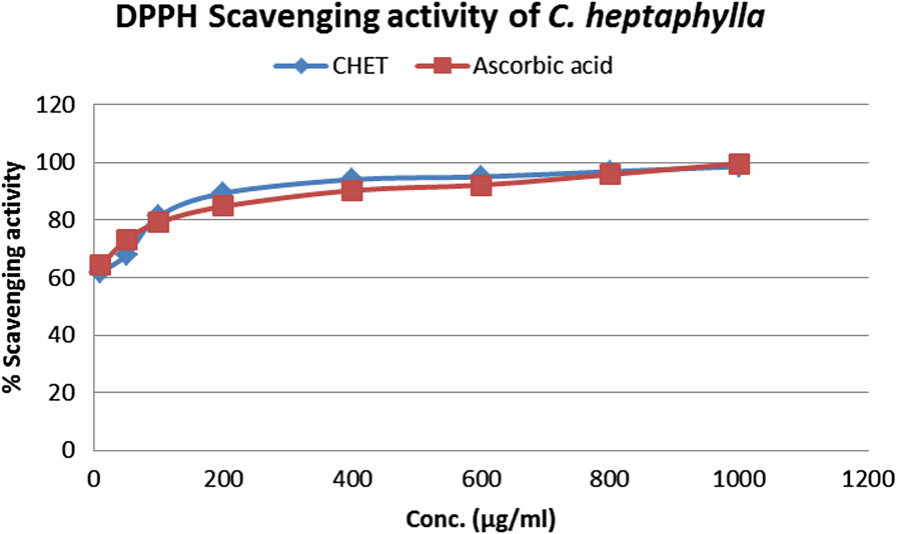
Relative percentage scavenging activity of standard antioxidant ascorbic acid and CHET extract.

The antioxidant activity of the extract was assessed by the DPPH free radical scavenging assay as shown in Figure 1. The stem bark extract exhibited significant DPPH free radical scavenging effects compared to standard ascorbic acid. Where, IC_50_ value of ascorbic acid was 5.15 μg/ml and stem bark extract was 3.11 μg/ml.

Antioxidant activity of *C. heptaphylla* extract was measured by DPPH free radical scavenging method and their scavenging activity was compared with the standard antioxidant ascorbic acid. Both ascorbic acid and extract showed a dose dependent activity. However, extract showed very strong DPPH free radical scavenging effect in regard to ascorbic acid. Among seven different concentrations used in the study (10 to 1000 μg/ml) 1000 μg/ml showed the highest scavenging activity 98.64% whereas ascorbic acid of the same concentration showed 99.65% which are very close to each other (Figure 1). Percent (%) scavenging activity was plotted against log concentration and from the graph IC_50_ (Inhibition concentration 50) value was calculated by linear regression analysis. IC_50_ value of ascorbic acid and extract was found 5.15 μg/ml and 3.11 μg/ml, respectively.

### Reducing power

The reducing power of a compound is related to its electron transfer ability and may therefore; serve as an indicator of its potential antioxidant activity [26]. By using the potassium ferricyanide reduction method the reductive capabilities of the plant extracts was identified in comparison with ascorbic acid. The reducing power of the extracts was moderately strong while increasing dose it shows little increment. At 100 μg/ml concentration, reducing power of CHET was 0.73 whereas that of ascorbic acid was 0.85. But, with increasing concentration (100-200 μg/ml), reducing power of CHET increased from 0.73 to o.89 whereas that of ascorbic acid increased from 0.85 to 1.54.

### Brine shrimp lethality bioassay

**Figure 2:**
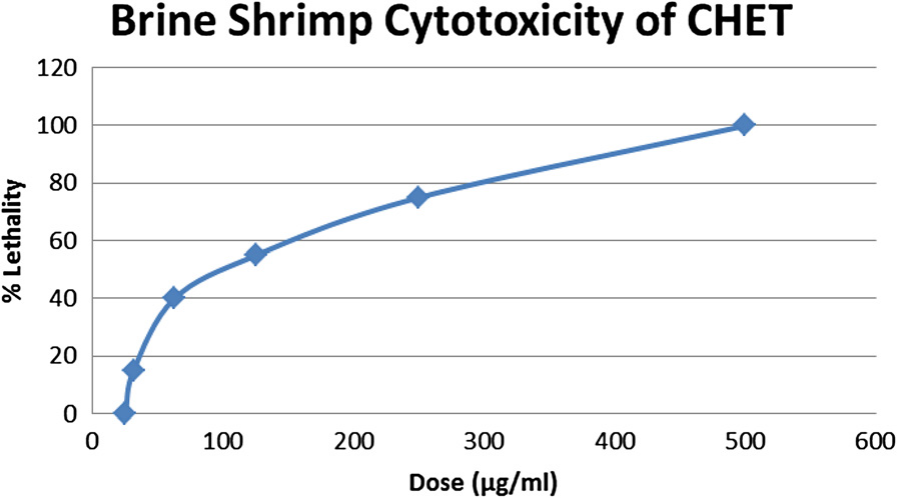
**Brine Shrimp Cytotoxicity of Ethanol Extract of *C. heptaphylla *(CHET).**

Cytotoxic effect of the extract is summarized in the Figure 2. In cytotoxic bioassay, the LC_50_ value of CHET was 144.1461 μg/ml.

### *In Vitro* clot lysis assay

**Figure 3:**
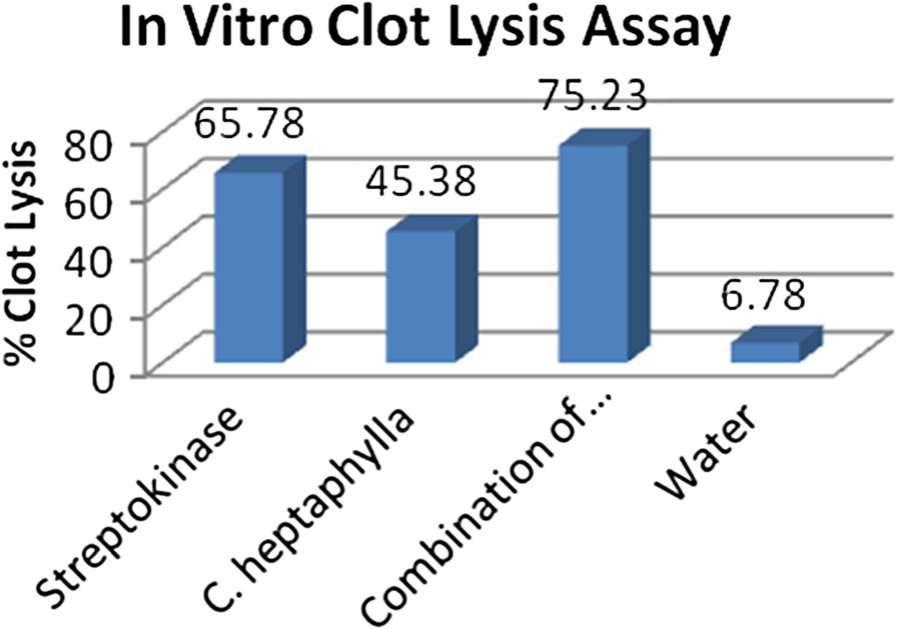
***In vitro *Clot lysis by Streptokinae, *Clausena heptaphylla*, *Clausena heptaphylla *in combination with Streptokinase and water.**

Addition of 100 μl Streptokinase (SK), a positive control (30,000 I.U.) to the clots along with 90 minutes of incubation at 37°C, showed 69.35 ± 1.88% clot lysis. Clots when treated with 100 μl sterile distilled water (negative control) showed only negligible clot lysis (6.23%). The mean difference in clot lysis percentage between positive and negative control was very significant (*p* < 0.0001). The *in vitro* thrombolytic activity study revealed that *Clausena heptaphylla* showed 44.64 ± 2.78% clot lysis and *Clausena heptaphylla* in combination with Streptokinase showed 74.23 ± 1.79% clot lysis activity. Percent clot lysis obtained after treating clots with herb and appropriate controls is shown in Figure 3. Statistical representation of the effective clot lysis percentage by our herbal preparation, positive thrombolytic control (Streptokinase) and negative control (sterile distilled water) is tabulated in Additional file 1: Table 3.

Statistical representation of the effective clot lysis percentage by herbal preparations, positive thrombolytic control (Streptokinase) and negative control (sterile distilled water) were done by paired t-test analysis; clot lysis % is represented as mean ± S.D. and *p* of herbal preparation (*C. heptaphylla*) was < 0.05; which was considered as significant.

### Antibacterial assay

CHET showed antibacterial activity as measured by zone of inhibition against all the bacteria tested except *Shigella sonnei*. CHET showed better antibacterial activity against gram-positive bacteria than gram-negative. Highest activity was demonstrated against *Enterococcus faecalis* and lowest against *Vibrio cholerae*. Again better antibacterial activity was demonstrated with 4 mg/disc concentration than 2 mg/disc concentration.

### Determination of MIC

The MIC value against Gram-positive and Gram-negative bacteria ranged from 2,000 to 3,500 μg/ml, respectively (Table 2). Lowest MIC value (2000 μg/ml) of CHET was against *Bacillus subtilis* and *Proteus* sp. MIC value was higher (3500 μg/ml) against *Klebsiella* sp., *Salmonella typhi* and *Shigella sonnei*. MIC value was much higher than control antibiotic, Doxycycline (30 μg/ml). Considering the impurities and complex composition of the extract, MIC value against the pathogens included in this study was promising though Doxycycline had much lower MIC value.

**Table 2:** Minimum Inhibitory Concentration of CHET and Doxycycline

**Test organism**		**Source ID (ATCC)**	**MIC value**	
			**CHET (μg/ml)**	**Doxycycline hydrochloride (μg/ml)**
Gram-positive	*Bacillus subtilis*	11774	2000	5.0
	*Staphylococcus aureus*	25923	2500	5.5
	*Bacillus cereus*	10876	2500	4.5
	*Bacillus polymyxa*	842	3000	6.0
	*Bacillus megaterium*	13578	3000	5.5
	*Enterococcus faecalis*	29212	3000	4.5
Gram-negative	*Salmonella typhi*	65154	3500	5.5
	*Shigella flexneri*	12022	3000	6.0
	*Klebsiella pneumoniae*	13883	3500	6.5
	*Shigella sonnei*	8992	3500	5.5
	*Proteus vulgaris*	13315	2000	4.5
	*E. coli*	25922	2500	5.0
	*Vibrio cholerae*	15748	3000	5.0
	*Pseudomonas aeruginosa*	27853	2500	4.5

### Antifungal assay

The anti-fungal potentials of the stem bark extract of *C. heptaphylla* were assessed against 5 fungal species. The results (diameter of zone of inhibition) were compared with the standard drug, Fluconazole (100 μg/disc). *C. heptaphylla* extract showed strong antifungal activity against the tested fungi (Table 3). All the tested fungi were susceptible to ethanolic stem bark extract of *C. heptaphylla.* CHET produced almost similar zone diameter compared with the standard drug in a very lower concentration (4-6 μg) compared to standard drug (100 μg).

**Table 3:** ***In-vitro *antifungal activity of CHET and fluconazole**

**Organism**	**Source**	**ID**	**% inhibition**		
			**CHET (4 mg)**	**CHET (6 mg)**	**Fluconazole (100 μg)**
*Aspergillus ustus*	DSM	63535	29.56	35.67	45
*Aspergillus niger*	DSM	737	37.87	41.98	65
*Aspergillus ochraceus*	DSM	824	39.98	43.66	41
*Penicillium chrysogenum*	DSM	1075	31.23	39.89	48
*Rhizopus oryzae*	DSM	2200	32.23	40.78	46

## Discussion

Plants produce a huge variety of secondary compounds as natural protection against microbial and insect attack. Some of these compounds are toxic to animals, but others may not be toxic. Indeed, many of these compounds have been used in the form of whole plants or plant extracts for food or medical applications in human [27] because plants are the natural reservoir of many antimicrobial, anticancer agents, analgesics, anti-diarrheal, antifungal as well as various therapeutic activities [28]. Acceptance of medicines from such plant origin as an alternative form of healthcare is increasing because they are serving as promising sources of novel antibiotic prototypes [29]. Some of the phytochemical compounds e.g. glycoside, saponin, tannin, flavonoids, terpenoid, alkaloids, have variously been reported to have antimicrobial activity [30].

Ethanolic stem bark extract of *Clausena heptaphylla* (CHET) contains flavonoids, alkaloids, saponins and steroids but it lacks tannins, anthraquinones & resins. Phenol content of the extract is 13.42 mg/g and flavonoid content is 68.9 mg/g.

Free radicals from oxidative stress are involved in many disorders like atherosclerosis, angina pectoris, neurodegenerative diseases and cancer. Antioxidants due to their scavenging activity are useful for the management of those diseases. The quantitative determination of antioxidants explored that high quantity of scavenging substances are found to be [31] in *C. heptaphylla* which plays the key role in showing free radical scavenging activity of this plant. CHET exhibited significant DPPH free radical scavenging activity with IC_50_ value of 3.11 μg/ml. Reducing power of CHET is also moderately strong.

Brine shrimp lethality is a general bioassay which is indicative of cytotoxicity, antibacterial activities, pesticidal effects and various pharmacologic actions [32]. In this research, six different concentrations (25, 31.25, 62.5, 125, 250, 500 μg/ml) of *C. heptaphylla* extract were used to determine its cytotoxicity by brine shrimp lethality bioassay (Figure 2). The extract showed lethality in a dose dependent manner. LC_50_ value of *C. heptaphylla* ethanol extract was found 144.1461 μg/ml at confidence limit 95% with chi-square value of 0.8533 (Figure 1). The LC_50_ value found in this study to be very significant suggesting the ethanol extract of *C. heptaphylla* has high potentiality to kill cancer cells as well as pests [32]. This significant lethality of the crude plant extract (as LC_50_ value less than 100 ppm or μg/ml) to brine shrimp is indicative of the presence of potent cytotoxic compounds which warrants further investigation.

*In vitro* thrombolytic activity of CHET is significant with 45.38% clot lysis capability compared to that of Streptokinase (65.78%) it can be considered for compound isolation in order to detect future anti-tumour compounds.

In antibacterial screening, moderate zone of inhibition (6.5-9.0 mm in diameter) were observed against gram-positive *Bacillus subtilis, Bacillus cereus, Staphylococcus aureus, Bacillus polymyxa* and *Bacillus megaterium* and less promising zone of inhibition (3.0-4.5 mm in diameter) against gram-negative *Salmonella typhi, Shigella flexneri,, Proteus sp.* and *Escherichia coli. Shigella sonnei* did not show any sensitivity*.* Stem bark extract showed significant zone of inhibition against *Rhizopus spp., Aspergillus niger and Aspergillus ochraceus* in antifungal assay*.*

CHET showed zone of inhibition to almost all the strains (at dose 2 mg and 4 mg/disc) except *Shigella soneii.* Crude extract at the concentration of 2mg/disc showed 5.5, 6.0, 5.9, 6.2 and 6.4 mm zone of inhibition diameter against Gram-positive *Bacillus subtilis, Staphylococcus aureus, Bacillus cereus, Bacillus polymyxa* and *Bacillus megaterium,* respectively and 3.1, 2.5, 1.8, 3.6 mm diameter against Gram-negative *Salmonella typhi, Shigella flexneri, Proteus sp.* and *E. coli*. On the other hand, standard antibiotic Kanamycin (30 μg/disc) showed significant antibacterial activity against all tested gram-positive and Gram-negative bacteria. Results implicated that the Gram-positive bacteria were more sensitive to the extract than the gram-negative bacteria. *Bacillus megaterium, Enterococcus faecalis, Staphylococcus aureusand Klebsiella* spp. were the most susceptible bacteria in this study (see Additional file 1). In a previous study, ethanolic stem bark extract of *Terminalia arjuna* showed 8-12 mm zone of inhibition diameter at 2 mg/disc concentration and 12-16 mm zone diameter at 4 mg/disc concentration against multi-antibiotic resistant *Vibrio cholerae*[33]. CHET has almost similar activity as *Terminalia arjuna* in this study. The present study justifies the claimed uses of *C. heptaphylla* in the traditional system of medicine to treat various infectious diseases caused by the microbes. The obtained results may provide a support to use of the plant in traditional medicine. Based on this, further chemical and pharmacological investigations to isolate and identify minor chemical constituents in *C. heptaphylla* and to screen other potential bioactivities may be recommended.

The MIC values against these bacteria were ranged from 2,000 to 3,500 μg/ml. The lowest MIC (2000 μg) was recorded against *Bacillus subtilis* and the highest MIC (3500 μg) recorded against *Salmonella typhi* and *Shigella sonnei.C. heptaphylla* showed a significant degree of anti-fungal activity (Table 2). The maximum anti-mycotic activity 43.66% was shown against *A. ochraceus*. Plant natural compounds are important source of mycotoxic compounds and they may provide a renewable source of useful fungicides that can be utilized in antimycotics drugs against infection of *A. ochraceus*. The effect of extract against *A. niger* was also higher implying that this plant can be utilized as anti-mycotics drugs against infection of *A. niger* in patients with pulmonary tuberculosis [34]. Moderate anti-mycotic effect was found against *Aspergillus ustus* at the concentration of 4 and 6 mg/ml. Fluconazole was used as standard antifungal agent to compare the potentials of extract (Table 3). There are, however, alarming reports of opportunistic fungal infections which describe that the resistance of the organisms increased due to indiscriminate use of commercial anti-microbial drugs commonly used for the treatment of infectious disease. This situation forced the researchers to search for new anti-microbial substance from various sources including medicinal plant [34]. Our research findings revealed that medicinal plant *C. heptaphylla* can play a vital role in combating fungal resistance.

## Conclusions

This study delineates that *C. heptaphylla* extract possesses some potentials in free-radical scavenging activity and cytotoxic effect but it has low antimicrobial (antibacterial and antifungal) activity. Since, crude ethanol extract of *C. heptaphylla* showed antibacterial and cytotoxic effect, it can be assumed that different active secondary metabolites were present in this extract. However, further studies are necessary to elucidate the mechanism lying with these effects. This study may serve as a foot step regarding the biological and pharmacological activities of stem bark extract of *C. heptaphylla.*

## Competing interests

The authors declare that they have no competing interests.

## Authors' contributions

MF planned the research work and collected the plant and drafted the manuscript. KSBM performed the antioxidant activity assay and *in vitro* thrombolytic assay. RMM has performed the antibacterial and antifungal and brine shrimp lethality assay. HA performed the phytochemical screening assay and reducing power assay. All authors read and approved the final manuscript.

## Pre-publication history

The pre-publication history for this paper can be accessed here:

http://www.biomedcentral.com/1472-6882/12/232/prepub

## Supplementary Material

Additional file 1 **Figure1.** Relative percentage scavenging activity of standard antioxidant ascorbic acid and CHET extract. **Figure 2:** Reducing potential of *C. heptaphylla.***Figure 3:** Probit analysis for brine shrimp treated with CHET. **Table 1:** DPPH Scavenging activity of *C. heptaphylla.***Table 2:** Brine Shrimp Cytotoxicity of Ethanol Extract of *C. heptaphylla.***Table 3+:** Effect of ethanolic stem bark extract of *C. heptaphylla* and streptokinase (Positive control) on *in vitro* clot lysis. **Table 4:***In-vitro* antibacterial activity of CHET and Kanamycin. Click here for file
