# Supplementary material for: Antibacterial, antifungal and antioxidant activities of the ethanol extract of the stem bark of Clausena heptaphylla
Source: BMC Complement Altern Med. 2012 Nov 27;12:232. doi: 10.1186/1472-6882-12-232 (PMC3533896; doi:10.1186/1472-6882-12-232)
Supplement: Additional file 1 — Figure1. Relative percentage scavenging activity of standard antioxidant ascorbic acid and CHET extract. Figure 2: Reducing potential of C. heptaphylla.Figure 3: Probit analysis for brine shrimp treated with CHET. Table 1: DPPH Scavenging activity of C. heptaphylla.Table 2: Brine Shrimp Cytotoxicity of Ethanol Extract of C. heptaphylla.Table 3+: Effect of ethanolic stem bark extract of C. heptaphylla and streptokinase (Positive control) on in vitro clot lysis. Table 4:In-vitro antibacterial activity of CHET and Kanamycin. [file 1472-6882-12-232-S1.doc]

**Supplementary data**

**Figures**

**Fig.1:** Relative percentage scavenging activity of standard antioxidant ascorbic acid and CHET extract

#

**Fig 2: Reducing potential of *C. heptaphylla***

**Fig. 3:** Probit analysis for brine shrimp treated with CHET.

# Tables

**Table 1:** DPPH Scavenging activity of *C. heptaphylla*

| **Sample Conc. (µg/ml)**  **Extract/AA** | **Log Con.** | **Absorbance** | | **% of scavenging activity** | | **IC_50_ (µg/ml)** | |
| --- | --- | --- | --- | --- | --- | --- | --- |
|  |  | **Extract** | **AA** | **Extract** | **AA** | **Extract** | **AA** |
| Control | - | 0.7925 | 0.7925 | - | - | 3.11 | 5.15 |
| 10 | 1.00 | 0.3011 | 0.2831 | 61.95 | 64.28 |  |  |
| 50 | 1.70 | 0.2531 | 0.2182 | 67.86 | 73.12 |  |  |
| 100 | 2.00 | 0.1567 | 0.1672 | 81.35 | 79.17 |  |  |
| 200 | 2.30 | 0.0845 | 0.1264 | 89.37 | 84.76 |  |  |
| 400 | 2.60 | 0.0502 | 0.0826 | 94.11 | 90.32 |  |  |
| 600 | 2.78 | 0.0452 | 0.0657 | 95.05 | 92.19 |  |  |
| 800 | 2.90 | 0.0285 | 0.0391 | 96.85 | 95.87 |  |  |
| 1000 | 3.00 | 0.0146 | 0.0117 | 98.64 | 99.65 |  |  |

***Table 2:*** *Brine Shrimp Cytotoxicity of Ethanol Extract of C. heptaphylla*

| Dose (µg/ml) | Log dose | total | alive | death | Lethality  % | Actual % | Probit | Probit (%) | Chi square |
| --- | --- | --- | --- | --- | --- | --- | --- | --- | --- |
| 25 | 1.398 | 20 | 20 | 0 | 0.0 | 0.0125 | 2.7582 | 0.0653 | 0.8533 |
| 31.25 | 1.495 | 20 | 17 | 3 | 15.0 | 0.15 | 3.9636 | 0.1019 | 0.455 |
| 62.5 | 1.796 | 20 | 12 | 8 | 40.0 | 0.40 | 4.7471 | 0.3006 | 0.6569 |
| 125 | 2.097 | 20 | 9 | 11 | 55.0 | 0.55 | 5.1254 | 0.5893 | 0.0525 |
| 250 | 2.398 | 20 | 5 | 15 | 75 | 0.75 | 5.6742 | 0.835 | 0.1732 |
| 500 | 2.699 | 20 | 0 | 20 | 100.0 | 0.9875 | 7.2418 | 0.9575 | 0.0188 |

Table 3: Effect of ethanolic stem bark extract of *C. heptaphylla* and streptokinase (Positive control) on *in vitro* clot lysis

| **Drug/ Extract** | **Mean ± S.D.**  **(clot lysis %)** | **t- value** | **P value**  **(two tailed) when compared to negative control (water)** |
| --- | --- | --- | --- |
| Streptokinase | 65.78± 1.46 | 102.76 | <0.0001 |
| *C. heptaphylla* | 45.38± 2.46 | 45.63 | <0.0001 |
| Combination of *C. heptaphylla* and Streptokinase | 75.23± 1.96 | 114.85 | <0.0001 |
| Water | 6.78± 1.06 | 11.34 | <0.0001 |

Table 4: *In-vitro* antibacterial activity of CHET and Kanamycin

| **Test organism** | | **Source ID**  **(ATCC)** | **Zone of inhibition (diameter in mm)** | | |
| --- | --- | --- | --- | --- | --- |
|  |  |  | **CHET**  **(2 mg/disc)** | **CHET**  **(4 mg/disc)** | **Kanamycin (30 µg/ disc)** |
| Gram positive | *Bacillus subtilis* | 11774 | 5.5 | 7.8 | 15 |
|  | *Staphylococcus aureus* | 25923 | 6.0 | 7.9 | 18 |
|  | *Bacillus cereus* | 10876 | 5.9 | 8.1 | 16 |
|  | *Bacillus polymyxa* | 842 | 6.2 | 8.2 | 17 |
|  | *Bacillus megaterium* | 13578 | 6.4 | 8.5 | 20 |
|  | *Enterococcus faecalis* | 29212 | 7.5 | 8.9 | 19 |
| Gram negative | *Salmonella typhi* | 65154 | 3.1 | 4.5 | 14 |
|  | *Klebsiella pneumonia* | 13883 | 6.9 | 8.6 | 18 |
|  | *Shigella flexneri* | 12022 | 2.5 | 3.2 | 15 |
|  | *Shigella sonnei* | 8992 | Nil | Nil | 14 |
|  | *Proteus vulgaris* | 13315 | 1.8 | 2.4 | 17 |
|  | *E. coli* | 25922 | 3.6 | 4.8 | 19 |
|  | *Vibrio cholerae* | 15748 | 1.2 | 2.1 | 16 |
|  | *Pseudomonas aeruginosa* | 27853 | 2.9 | 4.2 | 21 |
